# Screening for Preeclampsia in the First Trimester and Aspirin Prophylaxis: Our First Year

**DOI:** 10.1055/s-0040-1712124

**Published:** 2020-06-19

**Authors:** Inês Lourenço, Helena Gomes, Joana Ribeiro, Filipa Caeiro, Pedro Rocha, Carla Francisco

**Affiliations:** 1 Department of Gynecology and Obstetrics, Hospital Beatriz Ângelo, Loures, Portugal

**Keywords:** preeclampsia, first-trimester screening, aspirin, pré-eclâmpsia, rastreio primeiro trimestre, aspirina

## Abstract

**Objective** Preeclampsia is a major cause of perinatal and maternal morbidity and mortality. Our objective is to assess the performance of a combined screening test for preeclampsia in the first trimester and the prophylactic use of low-dose aspirin.

**Methods** Prospective study of all women attending our hospital for the first-trimester screening of aneuploidies, between March 2017 and February 2018 (*n* = 1,297). The exclusion criteria were multiple pregnancy and major fetal abnormalities. Preeclampsia screening was performed with an algorithm that includes maternal characteristics, and biophysical and biochemical biomarkers. High-risk was defined as a risk ≥ 1:50 of early-onset preeclampsia (before 34 weeks), in which cases low-dose aspirin (150 mg at night) was offered to these women from screening until 36 weeks.

**Results** From the 1,272 enrolled participants, the majority were Caucasian (1,051; 82.6%) and multiparous (658, 51.7%). Fifty patients (3.9%) screened high-risk for preeclampsia, and all started a low-dose aspirin regimen, with good compliance (96%). Early-onset preeclampsia was found in 3 pregnant women (0.24%), and total preeclampsia was diagnosed in 25 (2.02%), compared with 28 (0.75%) cases of early preeclampsia (*p* = 0.0099) and 98 (2.62%) of total preeclampsia (*p* = 0.2904) before the implementation of screening.

**Conclusion** There was a lower incidence of both, early-onset and total preeclampsia, after the introduction of universal screening and prophylactic use of low-dose aspirin. This reduction was statistically significant in early-onset preeclampsia. The association of a first-trimester combined screening model and aspirin prophylaxis appears to be useful in predicting and reducing the incidence of early-onset preeclampsia, in a routine care setting.

## Resumo

**Objetivo** A pré-eclâmpsia é uma causa importante de morbi-mortalidade materna e perinatal. Os objetivos do nosso estudo foram avaliar a implementação do rastreio combinado de pré-eclâmpsia no primeiro trimestre e o uso profilático de aspirina em baixa dose.

**Métodos** Estudo prospetivo das mulheres referenciadas ao nosso hospital para realização do rastreio do primeiro trimestre de aneuploidias, entre março de 2017 e fevereiro de 2018 (*n* = 1.297). Os critérios de exclusão foram gravidez múltipla e anomalias fetais graves. O algoritmo usado no rastreio da pré-eclâmpsia combina características maternas, e marcadores biofísicos e bioquímicos. Definiu-se alto risco como risco de pré-eclâmpsia precoce (antes das 34 semanas) ≥ 1:50, tendo sido recomendada aspirina em baixa dose (150 mg à noite) desde o rastreio até às 36 semanas.

**Resultados** Das 1.272 participantes, a maioria era caucasiana (1.051; 82,6%) e multípara (658; 51,7%). Cinquenta grávidas (3,9%) foram consideradas de alto risco para pré-eclâmpsia e todas iniciaram aspirina em baixa dose, com boa adesão (96%). Pré-eclampsia precoce foi diagnosticada em 3 grávidas (0,24%), e no total foram diagnosticados 25 casos de pré-eclâmpsia (2,02%), comparativamente com 28 (0,75%) casos de pré-eclampsia precoces (*p* = 0,0099) e 98 (2,62%) casos totais de pré-eclâmpsia (*p* = 0,2904) observados antes da implementação do rastreio. Verificou-se uma menor incidência de pré-eclâmpsia precoce e total após introdução do rastreio universal e uso profilático de aspirina. A redução da pré-eclâmpsia precoce foi estatisticamente significativa.

**Conclusão** A associação de um modelo de rastreio combinado no primeiro trimestre com o uso profilático de aspirina é aparentemente eficaz na redução do risco de pré-eclâmpsia precoce.

## Introduction

Preeclampsia (PE) affects 2 to 3% of all pregnancies, and, in developed countries, its incidence has increased in the last decades.^1^ ^2^ ^3^ ^4^ ^5^ Preeclampsia is considered one of the most important causes of maternal and perinatal morbidity and mortality.^1^ ^2^ ^3^ ^6^ ^7^ This condition is characterized by the development of new-onset high blood pressure after 20 weeks of gestation, with one or more of the following criteria: proteinuria, renal insufficiency, liver, neurological or hematological involvement, and fetal growth restriction.^8^ Preeclampsia is usually divided in early or late-onset preeclampsia, depending on whether the diagnosis is made before or after 34 weeks of gestation.^9^ ^10^ ^11^ This classification is directly related to the prognosis, as early-onset PE is associated with more severe complications and adverse maternal and perinatal outcomes.^6^ ^11^ ^12^ ^13^ ^14^ Early-onset PE has a lower incidence than late-onset PE, with a frequencies reported to be 0.5% and 1.4 to 1.7%, respectively.^5^ ^6^ ^14^ ^15^ Preeclampsia can also be classified as preterm or term, according to whether delivery is needed before or after 37 weeks of gestation.^1^ ^6^ ^16^

An impaired placentation is thought to be the underlying cause of PE, with complete resolution of the clinical manifestations within 12 weeks postpartum.^3^ ^12^ Some authors defend that the incomplete and defective trophoblastic invasion of the uterine spiral arteries, leading to uteroplacental hypoperfusion is the pathophysiologic mechanism responsible for the onset of PE.^3^ ^7^ ^13^ Furthermore, the presence of placental lesions associated with underperfusion is greater in early-onset PE, and these lesions are more severe in cases of very preterm delivery.^17^ ^18^ ^19^ The trophoblastic invasion of uterine arteries starts as early as 8 to 10 weeks and is completed around 16 to 18 weeks.^7^ Although the mechanism of action of aspirin in reducing PE is still unknown, some studies proposed that there is an improvement in uterine blood flow with a daily aspirin intake, suggesting it promotes the transformation of spiral uterine arteries into low resistance vessels.^7^ ^19^ Consequently, this pharmacologic intervention should reduce the prevalence of PE and minimize its complications.^6^ ^13^ ^19^ ^20^ Several studies proved that starting prophylaxis after 16 weeks of gestation was not associated with a significant improvement in outcome.^1^ ^7^ ^13^ ^21^ Moreover, a recent meta-analysis concluded that the administration of low-dose aspirin before 11 weeks had no significant effect in reducing PE.^22^ Taking this into consideration, the aspirin intake should start ideally between the 11^th^ and the 16^th^ weeks.^19^ ^22^ The aspirin for evidence-based preeclampsia prevention (ASPRE) trial validates these theories, as the aspirin subgroup had a significant reduction in the incidence of preterm PE when compared with the placebo subgroup.^6^ With regard to the optimal dose of aspirin, doses from 80 to 150 mg have proved to be effective.^19^ However, studies have shown a significant rate of aspirin resistance among pregnant women of ∼ 30%, 10%, and 5%, for doses of 81 mg, 121 mg, and 162 mg, respectively.^6^ ^19^ ^23^ ^24^ Considering that a prophylactic measure has shown to improve pregnancy outcomes, it is essential to have an effective screening method. Screening for PE in the first trimester allows the identification of high-risk pregnancies that will benefit from this intervention.^12^ The first-trimester combined screening for PE at 11 to 13 weeks of gestation uses an algorithm that includes maternal and pregnancy characteristics, such as biophysical markers—mean arterial pressure (MAP) and uterine artery pulsatility index (UtA PI)—and biochemical markers—serum levels of pregnancy-associated plasma protein A (PAPP-A) and placental growth factor (PLGF).^1^ ^13^ ^20^ According to a study published in 2013, this screening method is able to identify 95.3% of the early-onset PE cases and 45.5% of the late onset PE, using 1:200 as the cut-off risk, with a false positive rate of 10%.^20^ A different study revealed that screening by a combination of maternal characteristics, MAP, UtA PI, PAPP-A, and PLGF was able to identify 96.3% of the early-onset PE cases, using a cut-off of 1:269, with a 10% of false positives.^4^ The objective of our study was to assess the implementation of a combined screening approach for PE with the prophylactic use of low-dose aspirin in high-risk pregnancies.

## Methods

This is a prospective study of women attending our hospital for their routine first-trimester scan, in the first year of implementation of universal screening for preeclampsia, from the 1^st^ of March 2017 to the 28^th^ of February 2018.

### Population and Screening Method

All the women attending a first-trimester routine visit in our hospital were offered combined screening for PE, in addition to the routine screening for aneuploidies. At that first visit, between 9 and 11 weeks of gestation, data was collected on maternal characteristics, obstetric and medical history.^2^ Blood pressure was taken by validated automated devices, following a standardized protocol.^5^ ^13^ ^25^ Mean arterial pressure was measured twice in each arm and registered. In the same visit, maternal blood was taken to determine plasma levels of human chorionic gonadotrophin (HCG) and PAPP-A. At the first trimester ultrasound, between 11 weeks and 13 weeks and 6 days, gestational age was determined according to the fetal crown-rump length.^26^ Transabdominal color Doppler was used to measure the left and right UtA PI, and the average value was recorded.^5^ ^13^ ^27^ Women diagnosed with a multiple pregnancy or a major fetal abnormality were excluded. Maternal factors, and biophysical and biochemical markers (MAP, UtA PI, and PAPP-A) were combined by the software algorithm ViewPoint Version 5.6.12.601 (ViewPoint Bildverarbeitung GmbH, Wessling, Germany) and the risk of preeclampsia was calculated.^1^ ^4^ ^12^ ^13^ ^20^ In this study, high-risk was defined as a risk of early-onset PE ≥ 1:50. High-risk women were advised on their individual risk and offered low-dose aspirin, 150 mg every night, starting immediately after screening until 36 weeks of gestation. Women classified as high-risk were monitored in our hospital, in addition to their routine prenatal care, with follow-up scans at 22, 28, 32, and 36 weeks of gestation.

### Outcome Measures

The primary outcome was to determine the incidence of early-onset PE, defined as PE diagnosed before 34 weeks of gestation. The secondary outcome was to establish the incidence of total PE, defined as PE diagnosed at any gestational age. Preeclampsia was defined according to the International Society for the Study of Hypertension in Pregnancy.^8^ The compliance to aspirin was assessed by women's report of daily aspirin intake, during the follow-up prenatal visits.

### Statistical Analysis

All collected data were inserted into an Excel (Microsoft Corp., Redmond, WA, USA) database to perform a statistical analysis of maternal and pregnancy characteristics.

In each pregnant woman, the mean UtA PI, MAP, and PAPP-A serum levels were converted to multiples of the median (MoM), corrected for maternal and pregnancy characteristics.^4^ ^28^ The incidence of both early-onset PE and total PE were calculated, also in Excel. A Fisher exact test was used to compare the incidence of early-onset PE and total PE before and after the implementation of screening. Statistical significance was accepted at the level of *p* < 0.05.

## Results

### Population Characteristics and Screening

During the study's duration, a total of 1,297 pregnant women had their 1^st^ trimester ultrasound in our hospital. However, 25 of these women were excluded because they did not fulfill the eligibility criteria—22 had a twin pregnancy and 3 had a major fetal abnormality. Consequently, the screening for PE in the 1^st^ trimester was performed in 1,272 singleton pregnancies. The average maternal age of our population sample was 30 years. The majority was Caucasian (1,051; 82.6%), had a normal body mass index (BMI) (612; 48.1%), and did not smoke (1,091; 85.8%). Almost half of them were nulliparous (614; 48.3%), and 21 women had PE in a previous pregnancy (1.7%). The conception was spontaneous in 1,256 (98.7%), and 36 pregnant women (2.8%) had chronic hypertension. Table 1 describes the detailed maternal and pregnancy characteristics of this population.

**Table 1:** Maternal and pregnancy characteristics

Maternal and pregnancy characteristics	N = 1,272
Maternal age (years)	
Mean (±SD)	30.05 ± 5.9
Median [range]	30 [14–46]
< 25 years - nr. (%)	214 (16.8%)
25–35 years - nr. (%)	725 (57.0%)
≥ 35 years - nr. (%)	333 (26.2%)
Maternal BMI (Kg/m^2^)	
Mean (±SD)	25.06 ± 5.31
Median [range]	24 [15–53]
Normal BMI (18.5–25) - nr. (%)	612 (48.1%)
High BMI (≥25) - nr. (%)	596 (46.9%)
Low BMI (< 18.5) - nr. (%)	64 (5.0%)
Racial origin - nr. (%)	
Caucasian	1051 (82.6%)
Afro-Caribbean	161 (12.7%)
South Asian	31 (2.4%)
East Asian	4 (0.3%)
Mixed	25 (2.0%)
Cigarette smoking - nr. (%)	181 (14.2%)
Obstetric history - nr. (%)	
Nulliparous	614 (48.3%)
Multiparous without preeclampsia	637 (50.0%)
Multiparous with preeclampsia	21 (1.7%)
Medical history - nr. (%)	
Chronic hypertension	36 (2.8%)
Conception - nr. (%)	
Spontaneous	1,256 (98.7%)
Ovulation drugs or IVF	16 (1.3%)

Abbreviations: BMI, body mass index; IVF, in vitro fertilization; SD, standard deviation.

Of the 1,272 pregnant women that underwent PE screening, 50 (3.9%) screened positive for early-onset PE, and all of them started low-dose aspirin, 150 mg once per day at night. In the high-risk group, compared with low-risk group, the mean body mass index was higher and there was a higher prevalence of Afro-Caribbean origin, personal history of PE and chronic hypertension (Table 2).

**Table 2:** Maternal and pregnancy characteristics according to the preeclampsia screening risk group

Maternal and pregnancy characteristics	High-risk group (*n* = 50)	Low-risk group (*n* = 1,222)
Maternal age (years)		
Mean (±SD)	30.82 ± 6.7	30.03 ± 5.8
≥ 35 years - nr. (%)	17 (34%)	316 (25.9%)
Maternal BMI (Kg/m^2^)		
Mean (±SD)	27.04 ± 6.6	24.98 ± 5.2
High BMI (≥25) - nr. (%)	28 (56%)	568 (46.4%)
Racial origin - nr. (%)		
Caucasian	35 (70%)	1016 (83.1%)
Afro-Caribbean	14 (28%)	147 (12.1%)
Others	0 (0%)	35 (2.8%)
Mixed	1 (2%)	24 (2%)
Cigarette smoking - nr. (%)	6 (12%)	175 (14.3%)
Obstetric history - nr. (%)		
Nulliparous	30 (60%)	584 (47.8%)
Multiparous without preeclampsia	13 (26%)	624 (51.1%)
Multiparous with preeclampsia	7 (14%)	14 (1.1%)
Medical history - nr. (%)		
Chronic hypertension	18 (36%)	18 (1.5%)

Abbreviations: BMI, body mass index; SD, standard deviation.

### Pregnancy Outcomes

**Fig. 1:**
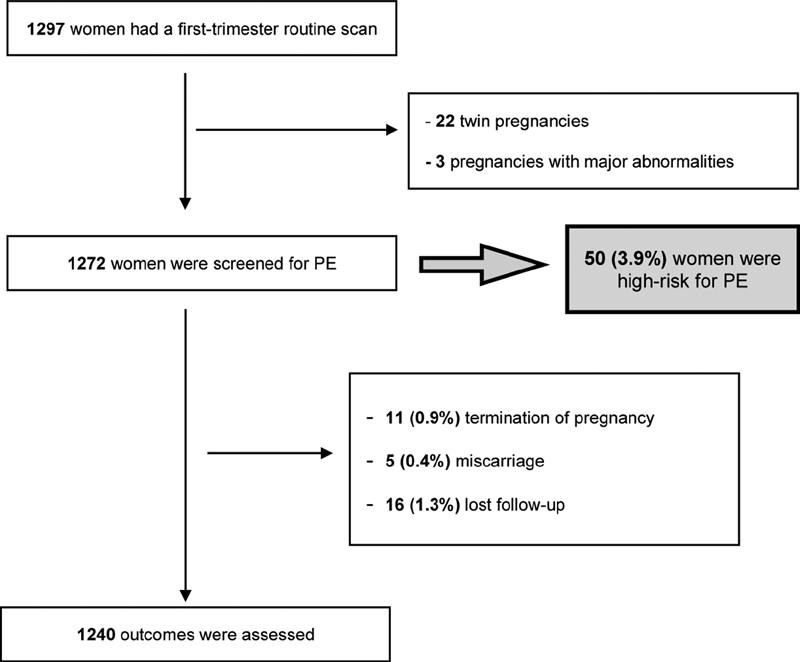
Population selection, screening, and follow-up.

Of the total women enrolled in PE first trimester screening, there were 11 terminations for fetal abnormalities (0.9%) and 5 miscarriages before 24 weeks of gestation (0.4%). Sixteen pregnant women were lost to follow-up (1.3%). Therefore, 1,240 pregnancies were included in the outcome assessment (Fig. 1).

### Primary Outcome

Early-onset PE occurred in 3 of the 1,240 pregnancies (0.24%) during our study period. This was compared with the incidence of early-onset PE observed in our hospital between 2014 and 2016, before the implementation of universal screening. During that period, early-onset PE was diagnosed in 28 of 3,747 women (0.75%), (*p* = 0.0099) (Table 3).

**Table 3:** Pregnancy outcomes

Pregnancy outcomes	Study period (1^st^ March 2017–28^th^ February 2018) n = 1,240	Before implementation of universal screening (2014–2016) n = 3,747	*P*-value
Primary outcome: diagnosis of PE before 34 weeks of gestation - nr. (%)	3 (0.24%)	28 (0.75%)	*p* = 0.0099
Secondary outcome: PE at any gestational age - nr. (%)	25 (2.02%)	98 (2.62%)	*p* = 0.2904

Abbreviation: PE, preeclampsia.

### Secondary Outcome

Total PE was diagnosed in 25 of 1,240 pregnancies (2.02%) in our study, compared with 98 of 3,747 (2.62%) observed before the implementation of screening (*p* = 0.2904) (Table 3). Of the total number of diagnosed PE, 3 cases were reported in the high-risk group and the other 22 in the low-risk group. Also, only 10 cases of PE required a preterm iatrogenic delivery (before 37 weeks of gestation).

## Compliance

The compliance to aspirin was good, as 48 (96%) of 50 high-risk women who started aspirin at the time of the screening maintained treatment until 36 weeks of gestation. The other 2 women (4%) stopped taking aspirin for intolerance.

## Discussion

In this prospective study, universal screening for PE was performed to all women attending our hospital for the first-trimester screening of aneuploidies. Based on a combined model, we identified high-risk pregnancies and started prophylactic aspirin at a dose of 150 mg per day, from 11 to 14 weeks of gestation until 36 weeks. The combined screening model used in our study, with an algorithm that considered maternal demographic characteristics, biophysical and biochemical biomarkers, has proved to be the most effective method of screening, with a high detection rate for early-onset PE.^1^ ^4^ ^12^ ^13^ ^20^ ^29^ The use of an early screening strategy allows the beginning of aspirin before the process of placentation is complete. This is in line with the results of several studies that suggest that the greater benefit of this prophylactic measure happens when it is started before 16 weeks.^7^ ^19^ ^21^ Early-onset PE was chosen as our primary outcome based on its clinical relevance, on the higher detection rate of the screening algorithm available and on the major benefit of aspirin in this subgroup, corroborated by several studies.^7^ ^17^ ^18^ ^21^ ^30^ The cut-off risk of 1:50 was selected considering the results from previous studies, as a compromise value to adapt the cut-off to our screening method and to our population, to have a high detection rate and a small number of false positives. Given the low rate of positive screen results (3.9%) in our study, we are considering revising our protocol and opt for a lower cut-off, to include more high-risk patients, in an attempt to reduce the rate of early-onset PE even further. As for the dosage selected in our study, we opted to use 150 mg of aspirin per day due to a known dose-dependent benefit and to reduce the aspirin resistance effect shown by recent evidence.^19^ ^23^ We also recommended that aspirin should be taken at night, as it is associated with a superior reduction of PE when compared with daytime administration.^19^ Our data showed a lower incidence of both early-onset PE and total PE when compared with the rates before the introduction of universal screening for PE. The reduction observed in early-onset PE was more substantial than the reduction seen in total PE, corroborating previous evidence available.^6^ ^7^ ^21^ ^30^ The reduction in the incidence of early-onset PE was statistically significant and probably meaningful to clinical practice. The performance of first-trimester screening is poorer for late-onset PE and low-dose aspirin has little or no beneficial effect in this condition.^4^ ^6^ ^20^ ^30^ The 3 cases of early-onset PE reported in the study occurred in the low-risk group, which probably means that no cases happened in the high-risk group because of the effect of aspirin prophylaxis, as the estimated number of cases in this group would be between 2 and 3. Moreover, this corroborates the importance of revising the cut-off to a more inclusive one. The strengths of our study are its prospective design, the screening model and prophylactic strategy are in line with the more recent evidence available and, as far as we know, it is the first study of PE screening and prophylaxis done in our country.^4^ ^6^ The main limitation is the small number of patients. We aim to continue PE screening in the future, obviating this limitation and strengthening the results.

## Conclusion

In conclusion, our study showed that the first-trimester screening of PE, which combines maternal factors, obstetric and medical history, biochemical and biophysical markers, is useful to predict early-onset PE in a routine care setting. Moreover, our results evidence a statistical reduction in the incidence of early-onset PE and also a small reduction of total PE, after the introduction of screening and prophylaxis. The prophylactic use of low-dose aspirin in high-risk pregnancies is most likely responsible for this reduction. However, further studies, with a larger population, are needed to corroborate these results.
